# A prospective phase II study of L-asparaginase- CHOP plus radiation in newly diagnosed extranodal NK/T-cell lymphoma, nasal type

**DOI:** 10.1186/1756-8722-6-44

**Published:** 2013-07-01

**Authors:** Ningjing Lin, Yuqin Song, Wen Zheng, Meifeng Tu, Yan Xie, Xiaopei Wang, Lingyan Ping, Zhitao Ying, Chen Zhang, Lijuan Deng, Weiping Liu, Jun Zhu

**Affiliations:** 1 Key laboratory of Carcinogenesis and Translational Research (Ministry of Education), Department of Lymphoma, Peking University Cancer Hospital & Institute, No. 52 Fucheng Road, Haidian district, Beijing 100142, China

## Abstract

**Purpose:**

To explore the efficacy and safety of L-asparaginase in newly-diagnosed extranodal nature killer (NK)/T –cell lymphoma (ENKTL), we conducted a prospective phase II study of L-asparaginase, cyclophosphamide, vincristine, doxorubicin and dexamethasone (CHOP-L) regimen in combination with radiotherapy.

**Patients and methods:**

Patients with newly diagnosed ENKTL and an ECOG performance status of 0 to 2 were eligible for enrollment. Treatment included 6–8 cycles of CHOP-L (cyclophosphamide, 750 mg/m^2^ day 1; vincristine, 1.4 mg/m^2^ day 1 (maximal dose 2 mg), doxorubicin 50 mg/m^2^ day 1; dexamethasone 10 mg days 1–8; L-asparaginase 6000 u/m^2^ days 2–8). Radiotherapy was scheduled after 4–6 cycles of CHOP-L regimen, depending on stage and primary anatomic site. The primary endpoint was complete response (CR) rate.

**Results:**

A total of 38 eligible patients were enrolled. The median age was 40.5 years (range, 15 to 71 years). Their clinical characteristics were male to female ratio, 24:14; Ann Arbor stage I, 20; II, 11; III, 3; IV, 4. CR and overall response rates were 81.6% (95% CI, 69.3% to 93.9%) and 84.2%, respectively. With a median follow-up of 25 months, the 2-year overall survival, progression-free survival and disease-free survival rates were 80.1% (95%CI, 73.3% to 86.9%), 81% (95%CI, 74.5% to 87.5%) and 93.6% (95%CI, 89.3% to 97.9%), respectively. The major adverse events were myelosuppression, liver dysfunction, and digestive tract toxicities. Grade 3 to 4 leukopenia and neutropenia were 76.3% and 84.2%, respectively. No treatment-related death was observed.

**Conclusion:**

CHOP-L chemotherapy in combination with radiotherapy is a safe and highly effective treatment for newly diagnosed ENKTL.

## Introduction

Extranodal NK/T-cell lymphoma, nasal type (ENKTL) is a highly aggressive and heterogeneous disease. It is much more prevalent in Asia than in Western countries. Its frequency among all malignant lymphoma is around 3%-10% in East Asia, but <1% in western countries [1,2]. Unlike that in Western countries, ENKTL is the most common peripheral T-cell lymphoma in China, with rate as high as 11% of all lymphoma [3,4]. Even though two-thirds of ENKTL patients present with localized disease, prognosis remains dismal [5]. Due to its rarity, it is difficult to do randomized, controlled trial. Therefore the best first-line chemotherapy regimen is not yet established.

Although radiation plays an important role in localized disease, treatment failure (including distant and loco-regional relapse) still occurs in nearly 50% of patients who are treated by radiation alone, and the 5-year survival rate ranges from 29.8% to 66% [6-9].

ENKTL tends to be resistant to conventional chemotherapy. Thus, it is imperative to develop an effective chemotherapy regimen. L-asparaginase, which is not regulated by multidrug resistance (MDR) gene, has been reported in relapsed or refractory ENKTL in retrospective studies with good response and survival rate [10-13]. Yong et al. first attempted to use L-asparaginase in refractory or relapsed ENKTL in 1990 in our center [14]. Subsequently a series of studies from our center [10,15-17] and two prospective studies [18,19] confirmed the remarkable efficacy of L-asparaginase in both newly- diagnosed stage IV and refractory/ relapsed ENKTL. These data suggested that L-asparaginase- based regimens represented a reasonable option in advanced, relapsed or refractory ENKTL [20,21].

To explore the value of L-asparaginase for newly diagnosed ENKTL, we conducted this prospective phase II study of L-asparaginase- based regimen in combination with radiotherapy. Based on our previous studies [10,16,17], we selected to combine L-asparaginase with cyclophosphamide, vincristine, doxorubicin and dexamethasone (CHOP-L). The design of the CHOP-L regimen was based on several considerations. Although some ENKTL showed resistance to CHOP regimen, 27% of patients still acquired CR from CHOP regimen [15]. Dexamethasone was used instead of prednisone to better prevent the adverse reactions of L-asparaginase [22].

## Patients and methods

### Eligibility Criteria

Patients of more than 14 years old with newly diagnosed ENKTL were eligible for CHOP-L phase II study, irrespective of stage and primary anatomic site. Eligibility criteria included biopsy- proven diagnosis of ENKTL, according to the World Health Organization (WHO) classification [23]. Tumor cells had a cytoplasmic CD3^+^, CD20^-^ phenotype, a cytotoxic profile and evidence of EBV infection (EBV- encoded small nuclear RNA by *in situ* hybridization). Most cases (35 among 38 with this marker available) were CD56+. The pathological diagnosis of all cases was confirmed by hematopathologists from our institution. Patients must have no prior chemotherapy or radiotherapy, an Eastern Cooperative Oncology Group (ECOG) performance status of 0 to 2, and a life expectancy of more than 3 months. Patients also had to have adequate bone marrow function (i.e., hemoglobin ≥80 g/l, absolute neutrophil count ≥ 1.0 × 10^9^/L, platelets ≥ 100 × 10^9^/L), renal function (i.e., serum creatinine ≤177 μmol/L),hepatic function (i.e., total bilirubin ≤ two times the upper limit of normal, and ALT /AST ≤2.5 times the upper limit of normal).

The exclusion criteria included CNS involvement, other concomitant malignant tumor, severe infection, pregnancy or breastfeeding, or psychiatric disorders. Patients were also excluded if they had a cardiac contraindication to doxorubicin therapy (e.g., abnormal contractility on echocardiography) or a neurologic contraindication to vincristine (e.g., peripheral neuropathy).

All patients gave their written informed consent before entering the study, which has been approved by the independent ethics committee of our institution.

### Study design and treatment

This is an open-label, single-arm prospective phase II study. The patients received 6–8 cycles of L-asparaginase, cyclophosphamide, vincristine, doxorubicin and dexamethasone (CHOP-L) given every 3 weeks. The dosage and administration schedule of CHOP-L were as follows: cyclophosphamide, 750 mg/m^2^ intravenously on day 1; vincristine, 1.4 mg/m^2^ intravenously on day 1 (maximal dose 2 mg); doxorubicin 50 mg/m^2^ intravenously on day 1; dexamethasone 10 mg intravenously on days 1–8; Escherichia coli L-asparaginase (Leunase, Kyowa Hakko Kogyo Co., Ltd, Tokyo, Japan; Changzhou Qianhong Bio-pharma Co., Ltd, Jiangsu, China) 6000 u/m^2^ intravenously on days 2–8.

Intradermal test was required prior to the administration of L-asparaginase. If intradermal test was positive or allergic reaction to L-asparaginase was observed, L-asparaginase was replaced by polyethylene glycosylated (PEG) - asparaginase (pegaspargase, Jiangsu Hengrui Medicine Co., Ltd., Jiangsu, China) 2500 u/m^2^ intramuscularly on day 2. During the treatment, patients were routinely monitored for coagulation function (including fibrinogen). Granulocyte colony- stimulation factor (G-CSF) was initiated when WBC count decreased to less than 2.0 × 10^9^/L or absolute neutrophil count ≤1.0 × 10^9^/L. G-CSF was discontinued when WBC count exceeded 10 × 10^9^/L or absolute neutrophil count ≥5.0 × 10^9^/L.

For upper aerodigestive tract NK/T lymphoma, patients with Ann Arbor stage I and II disease received involved- field radiation (IFRT) after 4–6 cycles CHOP-L regimen, and then finished remaining chemotherapy after completion of radiation; patients with Ann Arbor stage III and IV disease received IFRT for the primary anatomic site or residue lesion after finishing all chemotherapy. The time point of radiation was made at the discretion of treating physician. Three-dimensional conformal radiotherapy was done by using 6 MV photons generated from a linear accelerator at 2.0 Gy per daily fraction over 4–6 weeks.

### Staging

Baseline evaluation was performed within 2 weeks before enrollment. This consisted of a history taking, physical examination, routine blood tests including serum biochemistry with lactate dehydrogenase (LDH), bone marrow aspiration and biopsy, and lumber puncture. Computed tomography (CT), magnetic resonance imaging, and whole-body PET-CT scan with diagnostic quality CT, when possible, were used to evaluate stage, to assess the efficacy every 2 cycles, at the end of the study treatment, and to monitor relapse.

ENKTL was divided into two subtypes: upper aerodigestive tract NK/T- cell lymphoma (UNKTL) and extra- upper aerodigestive tract NK/T- cell lymphoma (EUNKTL). These two subtypes were defined in previous studies [24,25]. Lesion extending to adjacent organs/tissues was defined as Ann Arbor stage IE.

### Assessment

The primary endpoint was complete response (CR) rate. Secondary endpoints were overall response rate (RR), overall survival (OS), progression- free survival (PFS), disease- free survival (DFS) and toxicity.

Tumor responses were assessed every two cycles of chemotherapy, and were classified as complete response (CR), partial response (PR), stable disease (SD), or progressive disease (PD) according to the Revised Response Criteria for Lymphoma [26]. The interim assessment was made 2 weeks after the fourth cycle of the CHOP-L protocol, and the final assessment was made one month after the end of treatment.

All adverse reactions were graded each cycle according to the National Cancer Institute Common Toxicity Criteria, version 3.

### Statistical methods

#### Sample size

In our center, CR rate was 27% after CHOP regimen plus radiation for previously untreated ENKTL patients [15], and 55.6% with L-asparaginase, dexamethasone, plus vincristine for relapsed or refractory patients [10]. Therefore, a target CR rate of 56% with CHOP-L regimen was used to calculate the sample size. With a statistical power of 80% and a one-sided, type I error of 5%, the number of eligible patients required for this study was calculated to be 34. With an estimated dropout rate at 10%, the total sample size needed was 38 patients.

### Statistical analysis

OS was defined as the time from diagnosis to death from any cause or the date of last follow-up. PFS was measured from diagnosis to first progression, relapse after response, or death from any cause, or the date of last follow-up. DFS was calculated as the time from CR to death from any cause, relapse, or the date of last follow-up.

Data were analyzed using SPSS Statistics 16.0 software. OS, PFS and DFS were estimated using the Kaplan-Meier method. Survival curves were compared between groups using log-rank test. The impact of different factors on response was evaluated by chi-square test. *P* value less than 0.05 was considered statistically significant.

## Results

### Patient Characteristics

Between November 2008 and July 2012, a total of 38 Chinese patients (Mongoloid) were enrolled in this prospective study. Histologic diagnosis of all patients was confirmed as ENKTL by our central pathology review. The clinical characteristics of all patients are summarized in Table 1. Eighteen (47.4%) patients came from North China, 8 (21.1%) from the East coast region, 6 (15.8%) from Northeast China, 5 (13.2%) from Central China, and 1 (2.6%) from Southwest China.

**Table 1:** Clinical characteristics of patients with newly diagnosed ENKTL

**Characteristics**	**Patients**	
	**No**.	**%**
Gender		
Male	24	63.2%
Female	14	36.8%
Age, years		
>60	2	5.3%
≤60	36	94.7%
Ann Arbor Stage		
I	20	52.6%
II	11	28.9%
III	3	7.9%
IV	4	10.5%
Subtype of ENKTL		
UNKTL	36	94.7%
EUNKTL	2	5.3%
Tumor extending to adjacent organ/tissue in UNKTL	26 of 36	72.2%
Regional LN involvement	18	47.4%
Bony invasion or perforation	4	10.5%
B symptoms	22 of 38	57.9%
Fever	18 of 38	47.4%
Drenching night sweat	9 of 38	23.7%
Weigh loss	10 of 38	26.3%
ECOG performance status		
0	13	34.2%
1	24	63.2%
2	1	2.6%
IPI score		
0~1	27	71.1%
2	7	18.4%
3	2	5.3%
4~5	2	5.3%
NK/T-cell PI score		
0	10	26.3%
1	13	34.2%
2	7	18.4%
3~4	8	21.1%
Serum LDH elevated (>240 IU/L)	7	18.4%
ESR >30 mm/hr	10	26.3%
β2- microglobulin elevated	5	13.2%
Hemoglobin (<110 g/L)	2	5.3%
Leukocytopenia (<4.0 × 10^9^/L)	9	23.7%

Abbreviations: *UNKTL* Upper aerodigestive tract NK/T- cell lymphoma, *EUNKTL* Non-upper aerodigestive tract NK/T- cell lymphoma, *LN* lymph node, *ECOG* Eastern Cooperative Oncology Group, *IPI* International prognostic index, *NK*/*T*-*cell PI* NK/T-cell lymphoma prognostic index [27], *LDH* lactate dehydrogenase, *ESR* erythrocyte sedimentation rate.

There was a slight male predominance, with a male-to-female ratio of 1.7: 1. The median age was 40.5 years (range, 15–71 years), and 36 patients (94.7%) were less than 60 years old. The most frequent presenting symptoms were nasal obstruction (28/38, 73.7%),nasal mass (10/38, 26.3%),fever (5/38, 13.2%), pharyngalgia (4/38, 10.52%). The median time to diagnosis was 6 months (range, 1–48 months). 22 patients (57.9%) had B symptoms at presentation. Thirty six patients (94.7%) had upper aerodigestive tract NK/T-cell lymphoma (UNKTL), and only 2 were extra- upper aerodigestive tract NK/T-cell lymphoma (EUNKTL). Primary anatomic sites were naval cavity (n = 29), nasopharynx (n = 3), oropharynx (n = 4), coeliac lymph node (n = 1), and intestinal tract (n = 1). None of the patients had CNS or bone marrow involvement. 81.6% of the patients (31/38) had localized disease (Ann Arbor stage I/II). Among the 20 UNKTL patients with Ann Arbor stage I, 14 (70%) had contiguous extension to the adjacent organs/tissues (i.e., paranasal sinus, Waldeyer’s ring, bone, skin). HBs-Ag was negative in all patients, and anti-HBc Ab was positive in 11 patients. Platelets count and serum albumin level were normal in all patients.

### Treatment

All the patients received the scheduled treatment. The total cycles of CHOP-L regimen received by all patients were 193, with a median of 5 cycles (range, 2–8 cycles). Seven patients who rapidly progressed only received 2–4 cycles of CHOP-L. Because of positive intradermal test or allergic reaction related to L-asparaginase, 11 patients (28.9%) used pegaspargase instead of L-asparaginase in a total of 28 cycles, which accounted for 14.5% of all CHOP-L cycles, and the median cycle was 2 (range, 1–5 cycles).

In the 36 UNKTL patients, 31 received radical radiotherapy (RT). Two patients acquired CR after 4 or 8 cycles of CHOP-L regimen, and then rejected RT in fear of its toxicity. Three patients had no chance to receive RT due to rapid progression. The median radiation dose was 52 Gy (range, 40-60 Gy), and only two patients’ dosage was less than 50 Gy. 14 patients received RT after the end of chemotherapy (CT), and the other 17 patients received sandwich-like chemoradiotherapy (i.e., they received RT following 4 cycles of CHOP-L protocols, and then finished the remaining CHOP-L).

### Response

**Figure 1:**
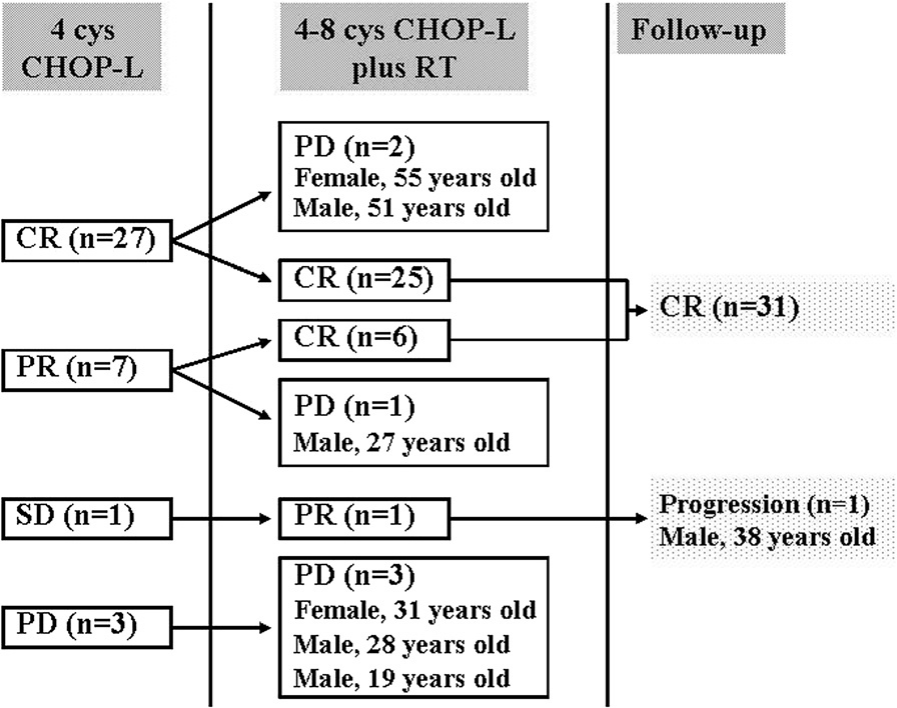
Summary of treatment scheme and outcomes.

The efficacy was estimated in all the 38 patients who received the scheduled treatment. After 4 cycles of CHOP-L regimen, interim assessment showed that there were 27 patients with CR (71.5%), 7 with PR (18.4%), 1 with SD (2.6%), and 3 with PD (7.9%). At the completion of all the chemoradiotherapy, 25 remained in CR, and 6 patients with PR converted to CR (Figure 1). Therefore 81.6% (31/38) of the patients achieved CR, with one PR (2.6%), and six PD (15.8%) after CHOP-L in combination with RT. The overall response rate (ORR) was 84.2%.

Among all the clinical factors, several had significant effects on CR status (*p*<0.05), specifically Ann Arbor stage (*p* =0.026) , B symptoms (*p* =0.014), ECOG score (*p* =0.013), IPI (*p* =0.001), NK/T-cell PI [27](*p* =0.001), LDH (*p* =0.013), WBC (*p* =0.004). Others factors, including age, gender, regional lymph node involvement, adjacent organ/tissue involvement in UNKTL, ESR, β2- microglobulin, hemoglobin and pegaspargase usage did not have significant effect on CR rate (Table 2).

**Table 2:** **Clinical characteristics and response rate after CHOP**-**L regimen plus radiation**

**Characteristics**	**N (%)**	**Therapeutic efficacy**		***P*value**
		**CR**	**No CR**	
Gender				1.000
Male	24(63.2%)	19(79.2%)	5(20.8%)	
Female	14(36.8%)	12(85.7%)	2(14.3%)	
Ann Arbor Stage				**0**.**026**
I	20(52.6%)	18(90%)	2(10%)	
II	11(28.9%)	10(90.9%)	1(9.1%)	
III	3(7.9%)	2(66.7%)	1(33.3%)	
IV	4(10.5%)	1(25%)	3(75%)	
Tumor extending to adjacent organ/tissue in UNKTL				0.157
Yes	26(72.2%)	20(76.9%)	6(23.1%)	
No	10(27.8%)	10(100%)	0(0%)	
Region LN involvement				0.222
Yes	18(47.4%)	13(72.2%)	5(27.8%)	
No	20(52.6%)	18(90%)	2(10%)	
Bony invasion or perforation				1.000
Yes	4(10.5%)	3(75%)	1(25%)	
No	34(89.5%)	28(82.4%)	6(17.6%)	
B symptoms				**0**.**014**
Yes	22(57.9%)	15(68.2%)	7(31.8%)	
No	16(42.1%)	16(100%)	0(0%)	
ECOG performance status				**0**.**013**
0	13(34.2%)	13(100%)	0(0%)	
1	24(63.2%)	18(75%)	6(25%)	
2	1(2.6%)	0(0%)	1(100%)	
IPI score				**0**.**001**
0~1	27(71.1%)	26(96.3%)	1(3.7%)	
2	7(18.4%)	3(42.9%)	4(57.1%)	
3	2(5.3%)	1(50%)	1(50%)	
4~5	2(5.3%)	1(50%)	1(50%)	
NK/T-cell PI score				**0**.**001**
0	10(26.3%)	10(100%)	0(0%)	
1	13(34.2%)	12(92.3%)	1(7.7%)	
2	7(18.4%)	6(85.7%)	1(14.3%)	
3~4	8(21.1%)	3(37.5%)	5(62.5%)	
Serum LDH level				**0**.**013**
Elevated	7(18.4%)	3(42.9%)	4(57.1%)	
Normal	31(81.6%)	28(90.3%)	3(9.7%)	
ESR				0.650
>30 mm/hr	10(26.3%)	9(90%)	1(10%)	
≤30 mm/hr	28(73.7%)	22(78.6%)	6(21.4%)	
β2- microglobulin				1.000
Elevated	5(13.2%)	4(80%)	1(20%)	
Normal	33(86.8%)	27(81.8%)	6(18.2%)	
Hemoglobin				0.339
<110 g/L	2(5.3%)	1(50%)	1(50%)	
≥110 g/L	36(94.7%)	30(83.3%)	6(16.7%)	
White blood cell count				**0**.**004**
<4.0 × 10^9^/L	9(23.7%)	4(44.4%)	5(55.6%)	
≥4.0 × 10^9^/L	29(76.3%)	27(93.1%)	2(6.9%)	
Pegaspargase replacement of L-asparaginase				1.000
yes	11(28.9%)	9(81.8%)	2(18.2%)	
no	27(71.1%)	22(81.5%)	5(18.5%)	

### Survival

**Figure 2:**
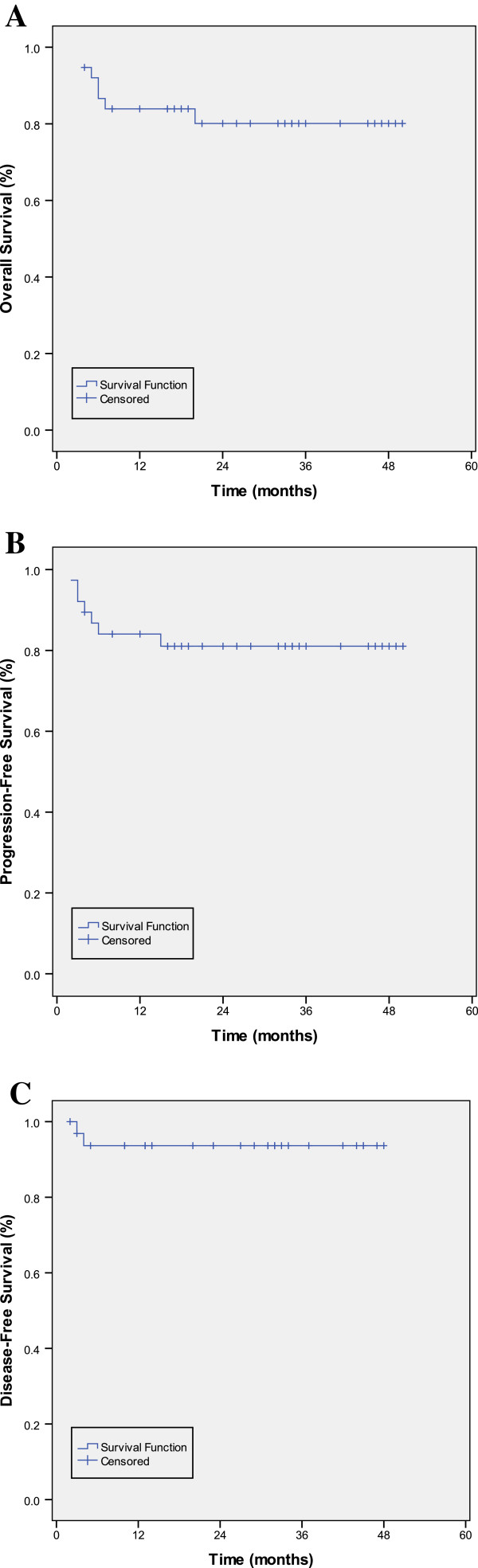
**The survival curves of 38 newly-diagnosed extranodal NK/T –cell lymphoma patients after CHOP-L plus radiation. (A)** Overall survival, **(B)** Progression- free survival, **(C)** Disease- free survival.

All of the 38 patients were eligible for survival analysis. The median follow-up for all patients was 25 months (range, 4–50 months). The median OS, DFS, and PFS points have not been reached. The 2-year OS, PFS and DFS rates were 80.1% (95%CI, 73.3% to 86.9%), 81% (95%CI, 74.5% to 87.5%) and 93.6% (95%CI, 89.3% to 97.9%), respectively (Figure 2).

During the follow-up period, seven patients died due to disease progression. Patients who achieved CR after treatment remained in CR to date, including the two UNKTL patients who declined radiation. One 38 year- old male patient with stage IIB at presentation achieved PR after treatment, and then received autologous hematopoietic stem cell transplantation (auto- HSCT). But six months later, the tumor progressed. This patient failed salvage chemotherapy and died of disease progression. His survival was 20 months. Another 27 year-old male with Ann Arbor stage IVB had primary refractory disease after 4 cycles of CHOP-L. He also died of systemic disease progression. Other five patients also had PD after 2–4 cycles of CHOP-L. They all died of lymphoma- associated hemophagocytic syndrome (HPS).

### Prognostic factors

In univariate analysis, the following clinical factors were significant poor prognostic factors for OS and PFS (*p* <0.05): Ann Arbor stage, B symptoms, ECOG score, IPI score, NK/T-cell PI score, LDH (>240 IU/L), leukopenia (<4.0 × 10^9^/L) and CR status. However, these factors except leukopenia were not significant for DFS. Other factors, including gender, tumor extending to adjacent organ/tissue in UNKTL, regional lymph node involvement, bony invasion or perforation, ESR >30 mm/hr, β2- microglobulin, hemoglobin<110 g/L and pegaspargase replacement of L-asparaginase had no significant effects on OS or PFS (Table 3).

**Table 3:** Univariate analysis of prognostic factors

**Factors**	***P*values**		
	**PFS**	**DFS**	**OS**
Gender	0.661	0.705	0.687
Ann Arbor Stage	**0**.**000**	0.079	**0**.**002**
Tumor extending to adjacent organ/tissue in UNKTL	/	0.354	0.116
Regional LN involvement	0.148	0.741	0.155
Bony invasion or perforation	0.735	0.643	0.752
B symptoms	**0**.**014**	0.170	**0**.**015**
ECOG performance status	**0**.**000**	0.260	**0**.**000**
IPI score	**0**.**001**	0.278	**0**.**002**
NK/T-cell PI score	**0**.**001**	0.291	**0**.**003**
LDH >240 IU/L	**0**.**001**	0.643	**0**.**002**
ESR >30 mm/hr	0.404	0.552	0.404
β2- microglobulin elevated	0.915	0.586	0.912
Hemoglobin <110 g/L	0.124	0.857	0.124
Leukopenia (<4.0 × 10^9^/L)	**0**.**000**	**0**.**002**	**0**.**001**
CR after treatment	**0**.**000**	/	**0**.**000**
Pegaspargase replacement	0.975	0.365	0.945

Note. The data with “bold fonts” emphasized that the *P* values were less than 0.05.

### Safety

The major adverse reactions to CHOP-L regimen were myelosuppression, liver dysfunction, coagulation dysfunction (decrease in fibrinogen) and digestive tract toxicities (Table 4). Grade 3 to 4 leukopenia and neutropenia were frequent (76.3% and 84.2%, respectively). Anemia and thrombocytopenia were mostly grade 1 to 2. Most patients (33/38, 86.8%) had a decrease in fibrinogen after L-asparaginase, but no increase in hemorrhage occurred. Other noticeable toxicities included hyperlipidemia, hypocalcemia, hypoglycemia and hyperglycemia (Table 4).

**Table 4:** **Toxicity profile of CHOP**-**L regimen**

	**Toxicity Incidence, No. (%)**				
**Toxicity**	**Grade 0**	**Grade 1**	**Grade 2**	**Grade3**	**Grade 4**
**Hematologic toxicity**					
Leukopenia	1(2.6%)	2(5.3%)	6(15.8%)	15(39.5%)	14(36.8%)
Neutropenia	1(2.6%)	2(5.3%)	3(7.9%)	9(23.7%)	23(60.5%)
Anemia	16(42.1%)	9(23.7%)	10(26.3%)	3(7.9%)	0
Thrombocytopenia	29(76.3%)	5(13.2%)	4(10.5%)	0	0
**Digestive tract effect**					
Nausea, vomiting	7(18.4%)	5(13.2%)	9(23.7%)	13(34.2%)	4(10.5%)
**Liver dysfunction**					
Increase in ALT/AST	7(18.4%)	15(39.5%	12(31.6%)	4(10.5%)	0
Increase in bilirubin	24(63.2%)	12(31.6%)	2(5.3%)	0	0
**Increase in BUN**	32(84.2%)	6(15.8%)	0	0	0
**Hypoalbuminemia**	16(42.1%), the minimum value was 27.9 g/L				
**Decrease in FIB**	33(86.8%), the minimum value was 55.2 mg/dL				
**Hypertriglyceridemia**	8(21.1%), the maximum value was 16.04 mmol/L				
**Hypercholesterolemia**	9(23.7%), the maximum value was 11.31 mmol/L				
**Hypocalcemia**	5(13.2%), the minimum value was 1.11 mmol/L				
**Hypoglycemia**	6(15.8%)				
**Hyperglycemia**	2(5.3%)				
**Changes in EKG**	9(23.7%)				
**Lung injury**	5(13.2%), 3 with grade 1, 2 with grade 2				
**Febril neutropenia**	8(21.1%)				
**Infection with grade 3 to 4 neutropenia**	4(10.5%)				
**Infection without neutropenia**	1(2.6%)				

Abbreviations: *BUN* blood urea nitrogen, *FIB* fibrinogen.

Nine patients (23.7%) had EKG abnormalities, including sinus tachycardia in six patients. The EKG changes had no effect on cardiac function or on chemotherapy schedules. Five patients (13.2%) had interstitial pneumonia, all recovered after corticosteroid treatment. Eight patients (21.1%) developed febrile neutropenia. One patient had allergic reaction to L-asparaginase infusion, and ten patients experienced a positive intradermal test after a median of 2 cycles of L-asparaginase (range, 1–5 cycles). No pancreatitis was seen. No treatment- related mortality was observed.

## Discussion

This study applied frontline treatment with L-asparaginase- based chemotherapy for ENKTL. Thirty- eight patients were recruited in this study. We excluded several types of patients with CNS involvement, other concomitant malignant tumor, severe infection, pregnancy or breastfeeding, or psychiatric disorders, because those situations would affect the proceeding of CHOP-L chemotherapy. This study indicated that the efficacy of L-asparaginase- based regimen (CHOP-L) in combination with radiotherapy was excellent for newly-diagnosed ENKTL. At the completion of treatment, the CR rate was 81.6%, which far exceeded the targeted CR rate of 56%. When we divided 36 upper aerodigestive tract NK/T- cell lymphoma (UNKTL) patients into localized group (Ann Arbor stage I/II) and advanced group (Ann Arbor stage III/IV), we found that CR rate was 90% and 50% in these two groups, respectively. The 2-yr OS and 2-yr PFS were 88.3%% and 89.5% in localized group, 50% and 50% in advanced group, respectively. Even though 18.4% of the cases recruited in our study were at Ann Arbor stage III/IV, the CR rate and survival were similar or superior to those reported from two concurrent chemo-radiotherapy studies (CCRT) for localized disease [28,29] (Table 5), and much better than historical data using anthracycline- based chemotherapy plus radiotherapy [15,30,31]. The CR rate for those ENKTL patients at advanced stage in our study was 42.9% (3/7), similar to those treated with SMILE regimen [19].

**Table 5:** Study comparison with recent prospective studies of ENKTL

**Author, year, country**	**Disease status**	**Study period**	**No. of patients**	**Treat**	**RT (Gy) Med. (Ran.)**	**CR rate**	**Survival**	
							**OS**	**PFS**
Lin et al. 2013, China (this study)	Newly- diagnosed	2008- 2012	Total cohort: 38	CHOP-L ± RT	52 (40–60)	81.6%	80.1% (2 yr-)	81% (2 yr-)
			UNKTL: 36					
			stage I/II: 30			90%	88.3%	89.5%
			stage III/IV: 6			50%	50%	50%
Kim et al. 2009, Korea	Newly- diagnosed, stage I/II, UNKTL	2006- 2007	30	CCRT + VIPD	40(40–52.8)	80%	86.3% (3 yr-)	85.2% (3 yr-)
Yamaguchi et al. 2009, Japan	Newly- diagnosed, stage I/II, UNKTL	2003- 2006	27	CCRT + DeVIC	50: stage I, 50.4: stage II	77%	78% (2 yr-)	67% (2 yr-)
Jaccard et al. 2011, France	Refractory, relapsed	2006- 2008	19	AspaMetDex	/	61%	Median OS: 12.2 months	
Yamaguchi et al. 2011 Japan	Newly- diagnosed stage IV, Refractory, relapsed	2007- 2009	38	SMILE	/	45%	55% (1 yr-)	53% (1 yr-)

Abbreviations: *No*. number, *RT* radiotherapy, *Med* Median; Ran, Range, *CR* complete remission, *OS*, overall survival, *PFS* progression-free survival, *yr* year, *UNKTL* Upper aerodigestive tract NK/T- cell lymphoma, *CCRT* concurrent chemoradiotherapy, *CHOP*-*L* cyclophosphamide, vincristine, doxorubicin, dexamethasone, and L-asparaginase, *VIPD* etoposide, ifosfamide, cisplatin, and dexamethasone, *DeVIC* dexamethasone, etoposide, ifosfamide, and carboplatin, *AspaMetDex*, L-asparaginase, methotrexate, and dexamethasone, *SMILE* methotrexate, ifosfamide, dexamethasone, etoposide, and L-asparaginase.

In our previous work, ENKTL patients treated with CHOP regimen followed by IFRT had a CR rate of 27% [15]. In a retrospective analysis of 77 cases of ENKTL [32], Li et al. found that anthracycline- containing chemotherapy (CT) alone resulted in worse response compared with radiation (RT) plus CT( CR, 50% vs. 74%; 5-year OS, 15% vs. 59%). There was also high incidence of locoregional and systemic failure (45% and 61%, respectively). In our study, interim assessment after 4 cycles of CHOP-L without RT demonstrated a CR rate of 71.5% (27/38). Therefore CHOP-L alone achieved high rate of disease-control comparable to CCRT (Table 5). This finding is very important in comparison to CCRT [33] because CCRT frequently caused serious mucosal toxicity, severe myelosuppression which led to delay of chemotherapy schedule.

Hypersensitivity reactions to L-asparaginase occurred in up to 30% of patients [34]. Recently, several studies confirmed that PEG-asparaginase had similar efficacy to L-asparaginase in childhood acute lymphoblastic leukemia [35,36]. In this study, 28.9% (11/38) of patients experienced an allergic reaction which happened after a median of 2 cycles of L-asparaginase chemotherapy (range 1–5 cycles). PEG-asparaginase was used in a total of 28 cycles, which accounted for 14.5% of all CHOP-L cycles, and the median cycles were 2 (range, 1-5 cycles). From statistically analysis, using PEG-asparaginase did not affect the CR rate, PFS, DFS and OS. PEG-asparaginase may become another good selection in the management of ENKTL. A multicenter prospective study is underway using PEG-asparaginase containing regimen in the treatment of newly diagnosed ENKTL in China.

Age, B symptoms, ECOG score, regional lymph node involvement, local tumor invasion, clinical stage, CR rate, lymphopenia, IPI and NK/T-cell PI were reported to have prognostic significance in ENKTL and T cell lymphoma [37,38]. In this study, univariate analysis showed that Ann Arbor stage, B symptoms, ECOG score, IPI score, NK/T-cell PI score, LDH (>240 IU/L), leukopenia (<4.0 × 10^9^/L) and CR were independent factors for OS and PFS in ENKTL. Leukopenia is frequently seen in ENKTL patients, and may cause delay of chemotherapy. In this study, leukopenia occurred in 23.7% (9/38) of patients who had no bone marrow involvement, and appeared to be associated with inferior survival. But regional lymph node involvement, tumor extending to adjacent organ/tissue in UNKTL and bony invasion or perforation did not affect the survival, possibly due to the high efficacy of L-asparaginase.

There were frequent grade 3 to 4 hematologic toxicities (76.3% for leukopenia and 84.2% for neutropenia) in this study, much higher than our previous study using L-asparaginase, vincristine plus dexamethasone in relapsed or refractory cases [10], in which 2.2% of cases experienced grade 3 to 4 leukopenia. Myelosuppression, nausea or vomiting, and liver dysfunction were more frequent than those in CHOP-21 regimen used in aggressive lymphoma [39,40], but much less than those occurring in SMILE study [19]. There was no treatment-related death in this study.

In conclusion, this prospective phase II study demonstrated that CHOP-L in combination with radiotherapy is a highly effective and well-tolerated treatment for patients with newly- diagnosed ENKTL.

## Competing interests

The authors declare that they have no competing interests.

## Authors’ contributions

All authors have contributed to data preparation, drafting and revising the manuscripts. All authors have read and approved the final manuscript.
